# From mean levels to localized peak concentrations: a province-level assessment of antibiotic pollution and ecological risk in Chinese surface waters

**DOI:** 10.1039/d6ra03099h

**Published:** 2026-07-03

**Authors:** Qiqi Zhou

**Affiliations:** a School of Biomedical and Environmental Engineering, Hefei Institute of Technology Hefei 238706 Anhui China zhouqq@hfit.edu.cn zhouq523@ustc.edu.cn

## Abstract

As the world's largest producer and consumer of antibiotics, China faces critical challenges regarding antibiotic pollution in its surface waters. Based on a provincial scale, this study systematically integrated literature data published from 2015 to 2025, providing a province-level synthesis of the spatial differentiation patterns, source characteristics, potential influencing factors, and peak-like concentration patterns of surface water antibiotic pollution across 34 provincial-level administrative units in China. Indicators including the median total antibiotic concentration, risk quotient (RQ), and maximum-to-mean (Max/Mean) ratio were employed to comprehensively analyze the spatial patterns, ecological risks, and occurrence characteristics. The results demonstrate a pronounced “east-high, west-low” spatial pattern for antibiotic pollution, with the average concentration in provinces east of the Hu Huanyong line (102 ng L^−1^) being approximately 2.4 times higher than that in western provinces (42 ng L^−1^). Fluoroquinolones (FQs) accounted for over 20% of the total concentration in 28 provinces, emerging as the dominant antibiotic class nationwide. Correlation analysis indicated that aquaculture intensity was positively associated with the environmental occurrence of aquaculture-use antibiotics, whereas the relationship between pig farming density and veterinary antibiotic load was positive but not statistically significant. High-risk provinces (average RQ ≥1) included Anhui, Guizhou, Shandong, Hebei, Hong Kong, Taiwan, and Macao, with ofloxacin, ciprofloxacin, and sulfamethoxazole identified as the primary high-risk substances. Notably, localized peak concentrations reaching up to 479.8 times the average concentration were observed in certain provinces. These peak-like cases were predominantly concentrated in scenarios such as aquaculture pond drainage (*e.g.*, Hubei and Shandong) and urban combined sewer overflows (CSOs, *e.g.*, Shanghai), suggesting potential acute ecological risks to aquatic ecosystems. This study provides systematic, provincial-scale evidence for comprehensively understanding antibiotic pollution in China. It is recommended that FQs be incorporated into the national priority monitoring list, differentiated control strategies be implemented, and environmental risk assessments be extended from traditional “mean-value control” to “pulse-fluctuation early warning.”

## Abstract 2

This study provides a province-level synthesis of antibiotic pollution and ecological risk in Chinese surface waters, highlighting localized peak concentrations that may be overlooked by conventional mean-based assessments.

## Graphical abstract

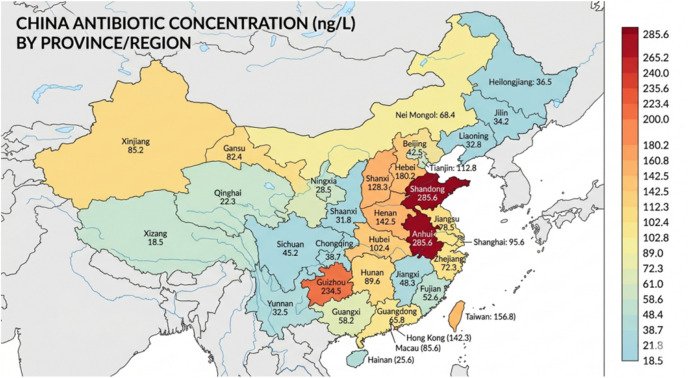

## 1 Introduction

With the rapid expansion of industrial, agricultural, and medical activities, antibiotics, as a typical class of contaminants of emerging concern (CECs), have been widely detected in aquatic ecosystems worldwide. Global annual antibiotic consumption has exhibited a surging trend, increasing from approximately 21.1 billion defined daily doses (DDDs) in 2000 to 34.8 billion DDDs in 2015, with China, India, and the United States being the largest consumers.^1,2^ Global antibiotic consumption is expected to continue increasing through 2030.^2^ Such continuously growing usage intensity has made the “pseudo-persistence” of antibiotics in the environment and their associated ecological and health risks a frontier hotspot in international environmental science.

In China, the massive production and consumption of antibiotics (*e.g.*, reaching a total usage of 162 000 tons in 2013, with human and veterinary uses each accounting for approximately 50%)^3^ have subjected the surface waters, sediments, and coastal waters of major river basins nationwide to severe pollution stress. Such contamination not only poses direct acute or chronic toxicity to aquatic flora and fauna—for instance, tetracyclines can inhibit the growth of green algae by interfering with chloroplast photosynthesis and protein synthesis, thereby impairing the reproductive capacity of aquatic organisms,^4^ while drugs like sulfadiazine can significantly alter microbial diversity and abundance^5^—but also presents a more profound threat. Specifically, they can significantly modify the diversity and functionality of benthic microbial communities and act as a potent chemical selective pressure, accelerating the evolution and dissemination of antibiotic resistance in the environment and profoundly impacting the “One Health” continuum.^5–7,10^

Existing studies indicate that antibiotic concentrations in Chinese river basins exhibit a pronounced “east-high, west-low” spatial pattern. Bounded by the “Hu Huanyong line,” the emission intensity in the eastern region is more than six times that in the west.^9,10^ Northern basins show significant antibiotic enrichment due to intensive agricultural irrigation and sluggish hydrological circulation, whereas southern basins are more predominantly influenced by intensive aquaculture and urban emissions.^11^ However, current macro-assessments of antibiotics at the national scale still have certain limitations. Existing environmental monitoring and risk assessment systems rely heavily on mean concentrations to characterize steady-state background pollution in river basins, paying insufficient attention to the spatiotemporal fluctuations and extreme peaks of pollution concentrations.^12^ In fact, driven by intermittent point-source emissions or abrupt hydrological events, episodic peak-like concentration fluctuations may occur in water bodies.^13^ Such instantaneous high-concentration shocks masked by mean values—such as storm wash-off, aquaculture pond drainage, and CSOs^14,15^—can elevate local antibiotic concentrations to tens or even hundreds of times the average level within a few hours, causing acute toxicological stress to aquatic organisms. Nevertheless, systematic quantitative analyses regarding the identification and underlying mechanisms of such events at the national scale remain lacking.^8^

Therefore, to overcome the limitations of traditional assessment frameworks and deeply elucidate the macroscopic occurrence characteristics and spatial driving mechanisms of antibiotics in Chinese water bodies, this study systematically reconstructed a provincial-scale antibiotic exposure mapping across the country. The core objectives of this paper are to:

(1) Comprehensively evaluate the overall residual levels of priority-controlled antibiotics in surface waters and identify the pollution signatures of key provinces and basins;

(2) Integrate ecological RQs with regional statistical factors to explore potential spatial associations between aquaculture/livestock activities and antibiotic occurrence patterns; and

(3) Introduce a maximum-to-mean (Max/Mean) ratio to quantify and identify localized peak concentrations in China's surface waters, thereby providing scientific support for shifting water environmental risk assessments from mean-value control to pulse-fluctuation early warning and implementing differentiated provincial management strategies.

### 1.1 Overview of the study area

The environmental occurrence and dissemination of antibiotics and antibiotic resistance are governed by complex climatic, landscape, and socioeconomic factors. Covering a vast land area of approximately 9.6 million square kilometers, China encompasses diverse climates ranging from cold-temperate to tropical. With a population of around 1.4 billion, the country is currently undergoing a period of rapid urbanization and socioeconomic transformation.

China's primary surface water systems comprise seven major river basins: the Yangtze, Yellow, Pearl, Songhua, Huai, Hai, and Liao rivers. Northern basins are characterized by water scarcity and low natural runoff, resulting in a limited dilution capacity for pollutants. Compounded by intensive agricultural and livestock activities, antibiotic enrichment in these regions is highly pronounced. In contrast, southern basins, which are heavily impacted by intensive aquaculture, urban discharges, and industrial wastewater, exhibit a wider variety of detected antibiotics at elevated concentrations. The antibiotic profiles in China's surface waters are predominantly characterized by sulfonamides, tetracyclines, and aminoglycosides. These contaminants primarily originate from agricultural non-point sources (*e.g.*, crop cultivation and livestock farming) and point source discharges (*e.g.*, effluents from wastewater treatment plants (WWTPs) and medical facilities).

By the end of 2024, national statistics recorded a total of 14 637 WWTPs, which processed 93.97 billion tons of wastewater annually, boasting a designed treatment capacity of 335 million tons per day.^16^ Although the urban wastewater treatment rate has exceeded 97%, the removal efficiency of conventional treatment processes for antibiotics remains highly limited. Consequently, effluents from WWTPs continue to act as a crucial, continuous source of antibiotics and antibiotic resistance genes (ARGs) in receiving waters.^17,18^

## 2 Materials and methods

### 2.1 Data sources and screening

A systematic literature search was conducted in the Web of Science, Scopus, and China National Knowledge Infrastructure (CNKI) databases to retrieve studies published between 2015 and 2025 concerning antibiotic pollution in China's surface waters. The search keywords included “antibiotics”, “surface water”, “river”, “lake”, “China”, and their corresponding Chinese equivalents. The inclusion criteria were defined as follows: (1) the study area must be located within China; (2) the sampling matrices must be surface waters (*e.g.*, rivers, lakes, reservoirs, wetlands, and estuaries); and (3) the study must provide at least one of the following metrics: antibiotic concentrations (mean or median), detection frequencies, or RQs. Conversely, the exclusion criteria were: (1) studies focusing exclusively on the influent/effluent of WWTPs or groundwater; (2) studies based on a single sampling site lacking spatial representativeness; and (3) studies that did not differentiate specific antibiotic compounds.

Ultimately, over 120 peer-reviewed articles were included, covering 34 provincial-level administrative units across China (including Taiwan, Hong Kong, and Macao). The extracted information comprised the province, water body name, sampling year, antibiotic name, concentration (maximum, minimum, median, and mean), detection frequency (%), RQ, and literature source.

To objectively reflect the actual pollution stress exerted on regional aquatic and terrestrial environments, spatial carrying capacity indicators were introduced in this study rather than directly employing absolute macroscopic production volumes. Specifically, the total number of slaughtered pigs and the total freshwater aquaculture production of each province in 2022 were divided by their corresponding provincial land areas. These values were then converted into pig breeding density (heads per km^2^) and freshwater aquaculture density (tons per km^2^), which served as the evaluation indicators for source emission intensity.

### 2.2 Calculation of antibiotic concentration indicators

To minimize the interference of extreme outliers and accurately reflect the typical pollution levels across provinces, the cumulative median concentration was adopted as the core indicator for spatial comparison. The specific calculation procedures were defined as follows:

(1) For each province, the representative median concentration of each individual antibiotic across all sampled water bodies was first determined. (In cases where multiple aggregated values were reported for the same antibiotic across different water bodies within a province, the overall median of these reported values was calculated to represent the provincial baseline);

(2) The representative median concentrations of all individual antibiotics within a given province were subsequently summed to derive the cumulative median concentration (ng L^−1^) for that province;

(3) If the median value for a specific antibiotic was unavailable in the literature, the reported mean value was utilized as a surrogate. Non-detect (ND) values were substituted with half the limit of detection (LOD/2). Data points lacking a specified LOD were excluded from the dataset to ensure data reliability. It should be noted that the cumulative median concentration used in this study was intended as a screening-level spatial metric for inter-provincial comparison, rather than a toxicity-weighted indicator or a measure of combined ecological risk. Because different antibiotics have distinct physicochemical properties, environmental persistence, and toxicological effects, the direct summation of median concentrations should be interpreted only as a generalized indicator of regional occurrence intensity. Compound-specific ecological risks were therefore further evaluated using the risk quotient (RQ) approach in Section 3.4.

### 2.3 Antibiotic categorization

The investigated antibiotics were grouped into five major classes according to their primary therapeutic applications and structural characteristics (Table 1).

**Table 1:** Classification and abbreviations of target antibiotics included in this study

Category	Class abbreviation	Target antibiotics	Compound abbreviation
Sulfonamides	SAs	Sulfamethoxazole	SMX
Sulfonamides	SAs	Sulfadiazine	SDZ
Sulfonamides	SAs	Sulfamethazine	SMZ
Sulfonamides	SAs	Sulfamethoxydiazine	SMR
Sulfonamides	SAs	Sulfamonomethoxine	SMM
Sulfonamides	SAs	Sulfaquinoxaline	SQX
Sulfonamides	SAs	Trimethoprim	TMP
Fluoroquinolones	FQs	Ofloxacin	OFL
Fluoroquinolones	FQs	Norfloxacin	NOR
Fluoroquinolones	FQs	Ciprofloxacin	CIP
Fluoroquinolones	FQs	Enrofloxacin	ENR
Fluoroquinolones	FQs	Levofloxacin	LEV
Fluoroquinolones	FQs	Lomefloxacin	LOM
Fluoroquinolones	FQs	Fleroxacin	FLE
Fluoroquinolones	FQs	Difloxacin	DIF
Fluoroquinolones	FQs	Sarafloxacin	SAR
Tetracyclines	TCs	Tetracycline	TC
Tetracyclines	TCs	Oxytetracycline	OTC
Tetracyclines	TCs	Chlortetracycline	CTC
Tetracyclines	TCs	Doxycycline	DOX
Macrolides	MLs	Erythromycin	ERY
Macrolides	MLs	Roxithromycin	ROX
Macrolides	MLs	Clarithromycin	CLA
Macrolides	MLs	Azithromycin	AZI
Macrolides	MLs	Tylosin	TYL
Others	—	Lincomycin	LIN
Others	—	Chloramphenicol	CAP
Others	—	Florfenicol	FF
Others	—	Amoxicillin	AMX
Others	—	Ampicillin	AMP
Others	—	Cefazolin	CFZ
Others	—	Cefmetazole	CFM
Others	—	Climbazole	CBZ
Others	—	Fluconazole	FCZ

## 3 Results and discussion

### 3.1 Spatial heterogeneity of antibiotic residues across 34 provinces

**Fig. 1:**
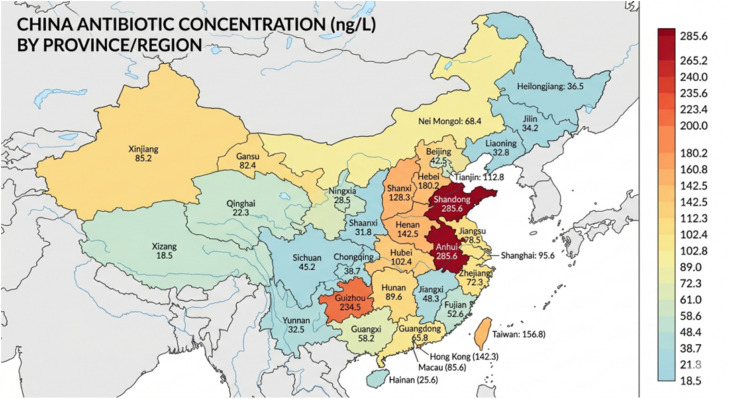
Spatial distribution of cumulative median concentrations of antibiotics in surface waters across Chinese provinces (ng L^−1^), based on aggregated literature data from 34 provincial-level administrative units.

The concentration of antibiotics in China's surface waters exhibited pronounced spatial heterogeneity (Fig. 1). The cumulative median concentrations across the provinces ranged from 18.5 to 285.6 ng L^−1^, with a national median of 64.1 ng L^−1^. Notably, the average concentration in provinces east of the Hu Huanyong line (102 ng L^−1^) was approximately 2.4 times higher than that in western provinces (42 ng L^−1^). This striking macroscopic contrast strongly aligns with the spatial distribution of intensive demographic and socioeconomic activities, highlighting anthropogenic pressure as a critical driver of spatial differentiation in pollution.

For analytical purposes, the studied provinces were categorized into three contamination tiers. High-pollution regions (>150 ng L^−1^) were primarily clustered in the North China Plain (*e.g.*, Shandong, Hebei), the Huai river basin (Anhui), and urban rivers in the southwest (Guizhou). These areas are predominantly characterized by intensive livestock breeding or high population density, while generally facing severe challenges of water scarcity or limited self-purification capacity. Moderate-pollution regions (50–150 ng L^−1^) encompassed the middle and lower reaches of the Yangtze River (*e.g.*, Hubei, Hunan, Jiangsu, Zhejiang), the Pearl River Delta (Guangdong, Macao), and certain inland provinces (Xinjiang, Gansu), reflecting the superimposed impacts of urban domestic sewage and industrial wastewater discharges. Low-pollution regions (<50 ng L^−1^) were mainly located in the Qinghai-Tibet Plateau (Tibet, Qinghai), Northeast China, and southwestern mountainous areas, where anthropogenic intensity is relatively low and water quality remains relatively pristine.

At the basin scale, the Huai river basin suffered the most severe contamination, followed by the Hai river basin, both exhibiting significant spatial variability. The Yangtze river basin displayed a distinct longitudinal gradient, with concentrations in the middle reaches surpassing those in the upper reaches. Similarly, the lower reaches of the Yellow River exhibited significantly higher concentrations than its upper segments. While the basins within the Qinghai-Tibet Plateau remained the least polluted regions nationwide, it is worth noting that localized hotspots were still identified within certain low-pollution provinces (*e.g.*, the Xining wetlands in Qinghai and the Lhasa River in Tibet). Given their high ecological fragility, these areas warrant incorporation into long-term monitoring frameworks.

Although the national median of the provincial cumulative concentrations in this study (64.1 ng L^−1^) may appear moderate in absolute terms, a comprehensive global comparison reveals its significant environmental implications. This value is approximately one order of magnitude higher than the stringent baseline levels typically maintained in highly regulated European river basins, where advanced wastewater treatment and strict pharmaceutical policies often suppress individual antibiotic residues to the sub-ng per L or low ng per L range.^19^ Conversely, this concentration falls squarely within the typical range reported for surface waters in rapidly industrializing and agricultural-intensive regions globally, while remaining substantially lower than the extreme µg per L-level pollution documented in localized manufacturing hotspots in South Asia.^20,21^ This intermediate positioning accurately reflects the complex “dual-pressure” scenario in China: immense total emission loads from intensive aquaculture and urbanization, partially mitigated by the progressive implementation of national environmental protection frameworks.

### 3.2 Compositional profiles of antibiotics across provinces

**Fig. 2:**
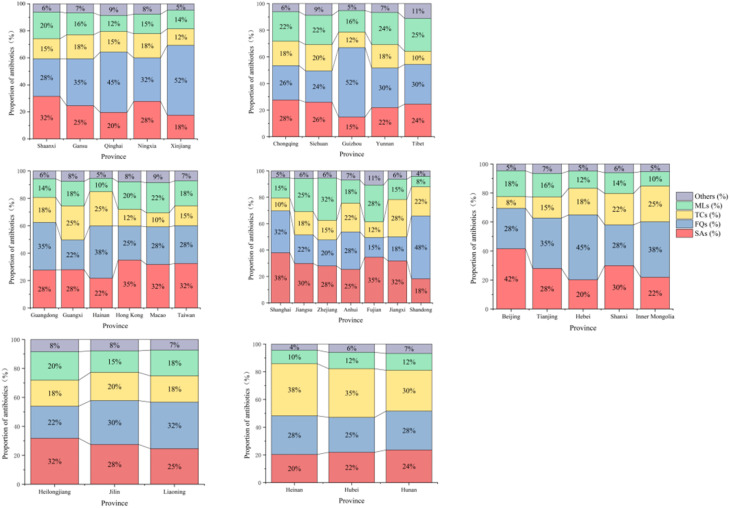
Compositional profiles of antibiotic classes across 34 provinces (percentage of median concentrations, %). (Note: percentages were calculated based on the median concentrations—or mean values when medians were unavailable—of each antibiotic class reported in the original literature. Potential sampling bias may exist in certain provinces, such as Tibet and Qinghai, due to limited data points. “Others” encompasses antibiotics not classified into the aforementioned four major categories, including β-lactams, lincosamides, chloramphenicols, and anthelmintics).

The compositional profiles of antibiotics in China's surface waters exhibit significant regional differentiation (Fig. 2). These spatial signatures are synergistically driven by urbanization levels, industrial structures, and geographical environments.

Distinct source characteristics exist within North China. As a megacity, Beijing is dominated by sulfonamides (SAs) and macrolides (MLs) (accounting for 59.3% collectively), exhibiting a typical urban WWTP-driven signature. In contrast, Hebei, Tianjin, and Inner Mongolia are co-dominated by fluoroquinolones (FQs) and tetracyclines (TCs) (53.1–63.1%), which is attributed to intensive livestock and poultry breeding. Notably, FQs account for 44.8% in Hebei, reflecting high-intensity emissions of veterinary antibiotics. Northeast China displays mixed contamination profiles, with Heilongjiang showing the highest proportion of SAs (31.8%). This may be related to the prolonged environmental half-life of SAs under low-temperature conditions, coupled with intensive domestic sewage discharges during the winter heating season in northern towns.^22,23^

East China presents a north-to-south evolution in its source profiles. As a major province for both the pharmaceutical industry and livestock breeding, Shandong shows a combined FQ and TC proportion of up to 69.7%, with its FQ share (47.6%) being the highest in East China, indicating robust mixed industrial-agricultural emissions. Moving southwards, economically developed regions such as Shanghai, Jiangsu, Zhejiang, and Fujian transition to SA- and ML-dominated profiles (53.1–62.4%), which is closely associated with the heavy domestic sewage loads from dense populations and highly developed pharmaceutical manufacturing. Central China (Henan, Hubei, Hunan) is the most prominent livestock-dominated region nationwide, with TCs and FQs collectively accounting for 57.5–65.5%. Specifically, the substantial proportion of TCs in Henan (37.6%) perfectly aligns with the antibiotic administration strategies in the large-scale swine and poultry farming industries of the central plains.

South China exhibits a dual characteristic of “high urbanization and intensive aquaculture.” Hong Kong, Macao, and Taiwan are dominated by SAs and MLs (52.0–55.1%), regulated by medical and domestic wastewater discharges. Conversely, Guangdong, Guangxi, and Hainan demonstrate strong FQ and TC signatures. The prominent FQ proportion in Hainan (38.1%) is hypothesized to stem from local tropical marine aquaculture and tropical animal husbandry. In Southwest China, Guizhou exhibits the highest national FQ proportion at 52.1%. Beyond urban sewage, this exceptional enrichment may be deeply tied to the unique hydrological structure of its karst landscapes—where frequent surface-groundwater interactions, impeded contaminant transport in karst fissures, and restricted photolysis facilitate the accumulation of highly adsorptive FQs.^24,25^ Although Tibet has lower absolute concentrations, its high ML proportion reflects localized domestic sewage pressure driven by plateau tourism.

Northwest China is characterized by the absolute dominance of FQs, with Xinjiang, Qinghai, and Gansu showing significant FQ proportions (32.2–51.7%). This is primarily governed by agricultural irrigation return flows from oases and the limited water dilution capacity in arid regions. Overall, FQs accounted for over 20% of the total antibiotic burden in 28 provinces nationwide. As a class of contaminants characterized by high biological toxicity and environmental persistence, their ubiquitous occurrence across the country warrants immediate and heightened attention from environmental management authorities.

### 3.3 Driving factor analysis

**Fig. 3:**
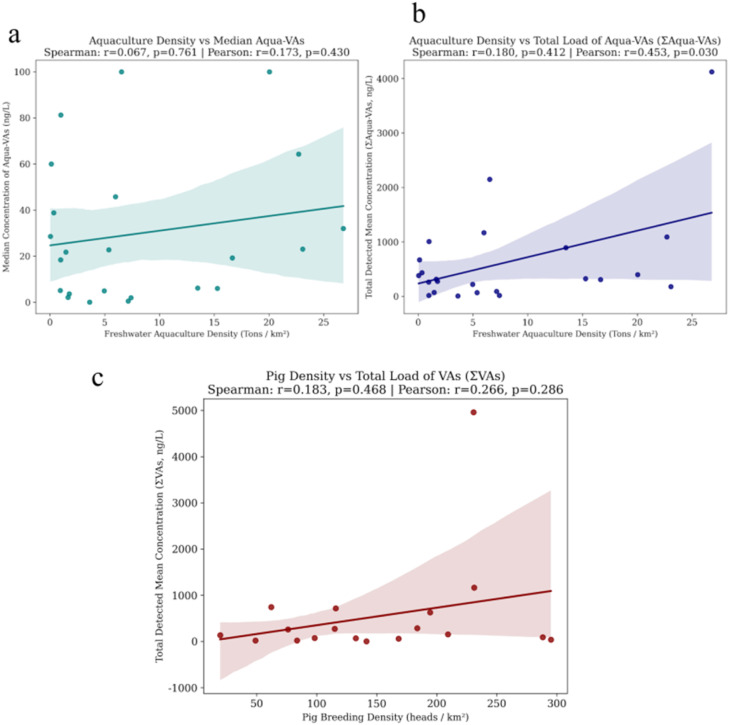
Correlation analysis between livestock and aquaculture production intensity and veterinary antibiotic concentrations in surface water across Chinese provinces. (a) Scatter plot of freshwater aquaculture density *versus* the median concentration of aquaculture-use antibiotics (Aqua-VAs). (b) Scatter plot of freshwater aquaculture density *versus* the total detection load of aquaculture-use antibiotics (ΣAqua-VAs). (c) Correlation analysis between pig farming density and the total detection load of veterinary antibiotics (VAs) in surface water. The sample sizes were *n* = 22 for aquaculture-related correlations (a and b) and *n* = 31 for the pig-farming-related correlation (c).

To explore potential large-scale spatial associations between regional production activities and antibiotic occurrence, spatial correlation analyses were conducted between regional breeding densities and the environmental occurrence of targeted antibiotics. As depicted in Fig. 3c, the total detection load of veterinary antibiotics (ΣVAs) exhibited a positive response trend to provincial pig breeding density. However, this correlation did not reach statistical significance (Pearson: *r* = 0.266, *p* = 0.286, *n* = 31). Rather than contradicting the emission inventory, this non-significant relationship indicates that although intensive swine farming constitutes a massive terrestrial pollution pool for VAs, their migration into surface water systems is strongly buffered by environmental interfaces. During the transport of terrestrial non-point source pollution across the land-water boundary, VAs undergo intense soil adsorption, photolysis, and interception by WWTPs. Consequently, a substantial fraction of VAs tends to accumulate at the water–soil interface rather than freely dissolving and diffusing into the bulk aqueous phase.

Conversely, the environmental response elicited by direct aquatic emissions presented a starkly different paradigm (Fig. 3b). When evaluating the correlation between freshwater aquaculture density and aquaculture-specific antibiotics (Aqua-VAs), the total environmental load (ΣAqua-VAs) demonstrated a significant positive correlation with aquaculture intensity (Pearson: *r* = 0.453, *p* < 0.05, *n* = 22). Unlike terrestrial livestock runoff, aquaculture pharmaceuticals are typically administered directly into the aquatic environment. Devoid of the interception provided by a soil buffering layer, unabsorbed Aqua-VAs can rapidly partition and enter the water body. Such point-source emission characteristics render regional surface water quality highly sensitive to localized aquaculture intensity.

The contrasting correlational outcomes between terrestrial and aquatic sources profoundly underscore the critical regulatory role of the water–soil interface in the environmental fate of emerging contaminants. For terrestrial emissions, constrained by strong interfacial partitioning effects, the conventional bulk water concentration is no longer an accurate proxy for the actual emission load. This implies that future ecological risk assessments and monitoring frameworks must shift their focus from the single bulk aqueous phase to the water–sediment (water–soil) interface. The intense accumulation of these emerging contaminants within such boundary microenvironments may exert sustained chemical pressure, triggering more profound and complex ecological consequences that warrant further targeted investigations.

It should be noted that these correlation analyses were based on aggregated provincial-level datasets and were intended to identify large-scale spatial association patterns rather than establish direct mechanistic causality. Potential confounding factors, including socioeconomic development, wastewater treatment infrastructure, hydrological conditions, and regional climatic variability, may also influence the observed patterns. Therefore, the reported relationships should be interpreted as exploratory associations. Future studies using standardized monitoring datasets and multivariate statistical approaches are needed to further quantify the relative contributions of different anthropogenic and environmental drivers.

### 3.4 Ecological risk assessment of targeted antibiotics in surface waters

**Fig. 4:**
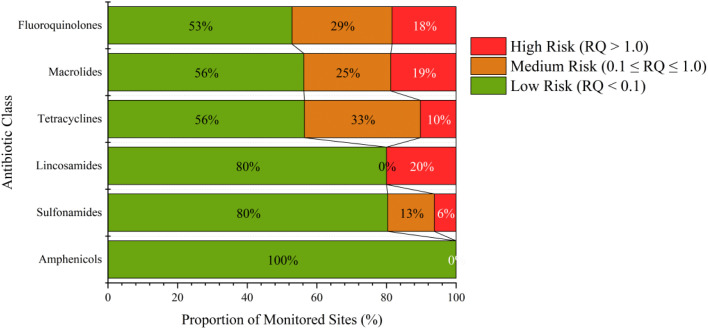
Ecological risk profiles of different antibiotic classes in Chinese surface water. The stacked bar chart illustrates the proportion of monitored sites categorized into three ecological risk tiers based on RQs: low risk (RQ < 0.1), medium risk (0.1 ≤ RQ ≤ 1.0), and high risk (RQ > 1.0).

To systematically evaluate the potential toxicological threats of antibiotic residues to aquatic ecosystems, this study conducted a hierarchical assessment of environmental risks for different classes of antibiotics based on the RQs (Fig. 4). The results indicate that the ecological risks of antibiotics in China's surface waters exhibit pronounced class-specific heterogeneity. The overall risks associated with sulfonamides and amphenicols remain manageable, with over 80% of the monitored sites demonstrating low risk (RQ < 0.1). This aligns with widely reported paradigms in the literature, which attribute this to the high susceptibility of these two antibiotic classes to photochemical degradation in the bulk aqueous phase and their tendency to be dominated by the physical dilution of basin hydrology.^26^

In stark contrast, FQs and MLs emerge as the primary drivers of ecological risk in China's surface waters. A staggering 18.4% of FQ and 18.8% of ML monitoring sites breached the high-risk threshold (RQ > 1.0), with nearly half of the sites falling into the moderate-to-high risk categories (Fig. 4). Multiple macro-level, in-depth investigations across national river basins (*e.g.*, the Yangtze and Pearl river basins) have also highlighted that MLs (*e.g.*, erythromycin, roxithromycin) and FQs (*e.g.*, enrofloxacin) exert highly specific inhibitory effects on chloroplast replication and protein synthesis pathways in aquatic primary producers, such as cyanobacteria and green algae. Compounded by continuous point-source inputs from high-density intensive aquaculture and municipal wastewater, these two classes of drugs are endowed with “pseudo-persistence” in the environment, thereby imposing long-term toxicological stress on aquatic biodiversity.^19^

More alarmingly, traditional RQ values calculated solely based on the bulk aqueous phase may underestimate the true ecological risks within the aquatic environment. Multiple recent authoritative studies have gradually shifted their focus from acute toxicity in the water phase to the selective pressure induced by chronic exposure concentrations. Driven by hydrophobic interactions and electrostatic attraction, FQs and MLs are highly prone to settling from the water phase and accumulating at the water–soil interface and within benthic sediments. Within such complex interfacial microenvironments, high concentrations of antibiotic residues may alter microbial community structures and exert selective pressure on microbial communities.

Although microplastics, ARGs, and water–sediment interfacial processes were not directly quantified in the present dataset, previous studies have suggested that these factors may provide important mechanistic perspectives for interpreting the environmental fate and potential ecological risks of antibiotics. As another class of emerging contaminants, microplastics may act as mobile interfacial carriers for antibiotic enrichment and microbial colonization. For example, FQs such as ciprofloxacin and norfloxacin often occur as zwitterionic species under environmentally relevant pH conditions, containing both acidic carboxyl groups and basic amino groups, which makes their sorption behavior highly sensitive to electrostatic interactions. Microplastic weathering can introduce oxygen-containing functional groups, such as carbonyl, hydroxyl, and carboxyl groups, onto microplastic surfaces, thereby enhancing their adsorption capacity for antibiotics, in some cases by up to 171%.^27^ Therefore, the binding affinity of certain antibiotics to microplastic surfaces may be enhanced, potentially increasing their persistence and mobility during aquatic transport. In addition, microplastic-associated biofilms have been reported to provide habitats for pathogenic and antibiotic-resistant bacteria, including *Vibrio*, *Salmonella*, and *Pseudomonas*.^27,28^ The co-occurrence of antibiotics, microorganisms, and ARGs at such micro-interfaces may increase local selective pressure and facilitate ARG enrichment and horizontal gene transfer under certain environmental conditions.^29,30^ Therefore, future studies and risk management strategies should move beyond aqueous-phase concentrations alone and further consider multi-media interfacial processes in antibiotic exposure and risk assessment.

### 3.5 Localized peak concentrations and potential acute ecological risks

**Fig. 5:**
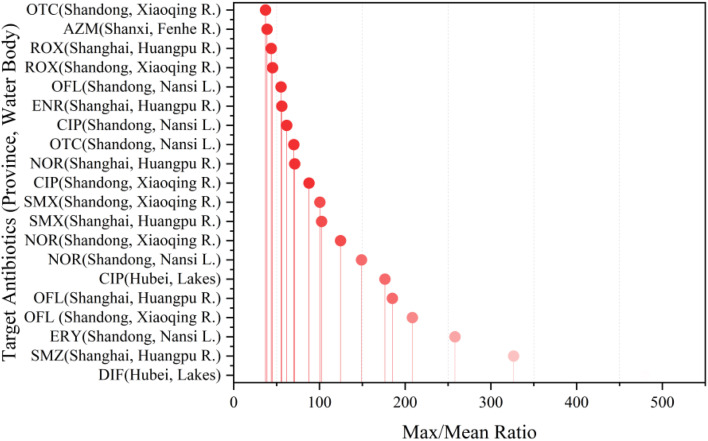
Identification of localized peak concentrations of targeted antibiotics in China's surface waters based on the Max/Mean concentration ratios.

While mean environmental concentrations are useful for characterizing chronic background pollution, they may overlook short-term or localized high-concentration occurrences that are relevant to acute ecological risk. In this study, the maximum-to-mean (Max/Mean) ratio was used as a screening-level indicator to identify potential concentration fluctuations or localized peak concentrations (Fig. 5). It should be noted that, because the dataset was compiled from heterogeneous literature sources with different sampling seasons, site selections, sampling frequencies, and analytical methods, high Max/Mean values should not be interpreted as direct evidence of temporally resolved pulse emissions. Instead, they represent potential worst-case exposure scenarios captured across different sampling campaigns.

For instance, DIF in typical lakes of Hubei and SMZ in the Huangpu River of Shanghai exhibited high Max/Mean ratios of 479.8 and 326.5, respectively. Similarly, the pulse multipliers for ENR and OFL in Lake Nansi and the Xiaoqing River of Shandong also breached the 200-fold mark. Such concentration spikes, spanning two to three orders of magnitude, may indicate episodic local inputs, sudden release of untreated effluents in localized waters, or cross-study variability.

Further spatial comparative analysis indicates that these localized peak concentrations are deeply and mechanistically coupled with regional dominant industries and land-use types.^31^ In major agricultural provinces characterized by intensive aquaculture and livestock breeding (*e.g.*, Hubei and Shandong), high Max/Mean events exceeding a factor of 100 were almost exclusively dominated by veterinary or aquaculture-specific drugs such as ENR and DIF. Extensive literature has corroborated that modern high-density intensive farming typically involves concentrated prophylactic drug administration during high-incidence periods of specific diseases. Subsequently, during pond drainage, water exchange, or heavy rainfall runoff, massive quantities of undegraded pharmaceuticals bypass environmental buffer zones and are discharged directly into receiving waters in a “pulse-like” manner. This provides a possible explanation for the episodic occurrence of startling pharmaceutical peaks in waters where the mean concentrations are otherwise extremely low.

Conversely, in the highly urbanized Huangpu river basin of Shanghai, the sudden surge of typical human/human-veterinary shared antibiotics, such as OFL (a 185-fold pulse), unveils a starkly different mechanism intertwined with urban point and non-point sources. In catchments dominated by urban impervious surfaces, extreme hydrological events like rainstorms are highly prone to triggering CSOs or the hydraulic overloading of WWTPs.^15^ Multiple in-depth investigations of municipal watersheds have highlighted CSOs as a core catalyst for the acute concentration spikes of medical and domestic sewage markers in urban surface waters. Additionally, the episodic leakage or spilling of effluents from surrounding pharmaceutical enterprises or medical institutions may also act as a crucial underlying factor driving the localized peaks of specific antibiotics (*e.g.*, SMZ).

Furthermore, the scientific significance of this stable baseline lies in its stark contrast to the episodic “short-term high-concentration exposures” identified in this study. It is precisely because the chronic background remains at a relatively low level that the localized peak concentration events—associated with agricultural pond drainage or urban CSOs and characterized by concentration spikes spanning two to three orders of magnitude—may contribute to potential acute ecological risks to aquatic ecosystems.^32,33^ These transient but severe fluctuations suggest that future ecological risk assessments should place greater emphasis on high-frequency monitoring of localized peak concentrations, rather than relying solely on steady-state mean-value baselines.

### 3.6 Limitations and management implications

#### 3.6.1 Limitations and future perspectives

Although this study comprehensively evaluated the macroscopic occurrence and exposure risks of antibiotics in surface waters nationwide, it remains constrained by the spatiotemporal heterogeneity of the available literature data. First, baseline and driving-factor data for the ecologically fragile central and western regions remain relatively scarce. Moreover, the varying sampling frequencies across different studies and the lack of long-term, continuous time-series monitoring make it difficult to accurately quantify the dynamic response of pollution loads to the interventions of national environmental policies, such as the Action Plan for Prevention and Control of Water Pollution (commonly known as the “Water Ten Plan”). Future efforts urgently need to rely on standardized national environmental monitoring networks to bridge these spatial data gaps and achieve high-frequency dynamic tracking. In addition, the Max/Mean ratio used in this study should be regarded as a screening-level indicator rather than direct evidence of real-time pulse emissions. Due to the heterogeneity of literature-derived datasets, the observed peak-like values may reflect both episodic local inputs and cross-study variability. Therefore, future studies based on standardized high-frequency monitoring are needed to verify the temporal dynamics and ecological consequences of such high-concentration occurrences.

Second, current source apportionment precision and single-substance risk assessments (*e.g.*, the RQ method) urgently require an upgrade toward complex system frameworks. Given the overlapping human and veterinary use of most antibiotics, refined techniques such as stable isotope analysis or microbial source tracking must be introduced in the future to decouple the precise contributions of mixed sources. More crucially, future risk assessments must break through the traditional single-exposure paradigm. They should not only incorporate elusive transformation products (TPs) but also focus on the combined toxicity of antibiotics and co-existing contaminants, such as microplastics and heavy metals. In particular, there is an urgent need to elucidate how these compound chemical stresses drive the enrichment of ARGs and horizontal gene transfer within benthic communities at the water–sediment micro-interface. Addressing these knowledge gaps will constitute the core frontier for constructing the next generation of basin-scale ecological health assessment systems.

#### 3.6.2 Management implications and control strategies

The high ecological risks and localized peak concentration characteristics of targeted antibiotics underscore the inherent limitations of the existing “mean-value control” and “end-of-pipe treatment” paradigms. To effectively mitigate surface water pollution, management frameworks must pivot toward “precise source tracking and event-driven early warning.” First, for agricultural and aquaculture basins exhibiting ultra-high pulses, it is imperative to promote the reduction of dedicated veterinary drugs and construct ecological buffer zones at the land-water ecotone to intercept non-point source runoff. Second, environmental monitoring networks should incorporate high-frequency pulse early warning mechanisms, focusing on the strict regulation of episodic direct discharges during high-risk periods, such as pond drainage and water exchange. Finally, in highly urbanized catchments, the retrofitting of CSOs and the construction of stormwater detention basins must be accelerated. This will curtail the instantaneous deluge of human-use antibiotics during rainstorms at the source, thereby preventing receiving waters from suffering potential acute ecological risks.

Furthermore, future environmental risk management frameworks should further consider the limitations of current singular “macroscopic aqueous phase assessments” and extend towards the microscopic mechanisms of multi-media complex interfaces. Although the present study suggests that baseline concentrations in flowing water bodies are generally at relatively moderate levels, antibiotics exhibit a strong propensity for phase partitioning and intense accumulation at the water–soil (or water–sediment) interface. Particularly within these boundary microenvironments, the widespread dispersion of novel vectors, such as microplastics, may enhance localized exposure concentrations through adsorption and biofilm-associated processes. The plastisphere—the biofilm forming on microplastic surfaces—acts as a potential retention hotspot for these residues.^34^ Furthermore, the sediment itself serves as a crucial environmental reservoir where extracellular DNA can persist for extended periods, significantly facilitating the propagation of ARGs.^35^ Consequently, even seemingly non-extreme macroscopic aqueous background values can exert sustained, selective pressure within these interfacial microzones, thereby potentially facilitating the enrichment and horizontal transfer of ARGs among benthic microbial communities.^36^ Incorporating the interfacial driving effects of multiple environmental factors into future exposure models may provide an important direction for improving future early-warning frameworks of antibiotic ecological risks.

## 4 Conclusions

This study provides a macroscopic, province-level synthesis of antibiotic occurrence and ecological risks in Chinese surface waters based on literature data published from 2015 to 2025. The results indicate that antibiotic contamination exhibits clear spatial heterogeneity, with higher overall occurrence levels in eastern and more intensively developed regions than in western regions. Fluoroquinolones (FQs) and macrolides (MLs) were identified as widely distributed and ecologically relevant antibiotic classes, suggesting that they deserve greater attention in future monitoring and risk management.

The correlation analyses further suggest that regional breeding and aquaculture activities may be associated with the spatial occurrence patterns of certain antibiotic groups. However, these relationships should be interpreted cautiously as exploratory spatial associations rather than direct causal mechanisms. In addition, the Max/Mean-based analysis highlights the value of considering localized peak concentrations that may be overlooked by traditional mean-based assessments. These peak-like concentration patterns provide a useful screening signal for identifying areas where short-term, high-concentration exposures may contribute to acute ecological risks.

Overall, the principal conclusions of this study emphasize the need to move antibiotic pollution control in China beyond uniform, mean-value-based assessments and toward more differentiated, region-specific monitoring strategies. Priority should be given to high-risk antibiotic classes, representative hotspot regions, and scenarios where localized peak concentrations frequently occur, such as agricultural drainage and urban runoff-related events. Finally, future studies utilizing standardized, high-frequency monitoring and multi-media datasets will be essential for comprehensively verifying temporal concentration dynamics, source contributions, and synergistic risk mechanisms at environmental micro-interfaces.

## Conflicts of interest

There are no conflicts to declare.

## Data Availability

All data generated or analyzed during this study, including the calculated median concentrations, pollution fingerprints, and Max/Mean pulse ratios, are included within the manuscript's figures and tables. The raw data used for this secondary analysis are derived from previously published studies, which are fully cited in the reference list. The compiled datasets are available from the corresponding author upon reasonable request.
